# New Kinase Inhibitors That Are Selectively Cytotoxic for Tumor Cells

**DOI:** 10.1134/S1607672924601446

**Published:** 2025-04-12

**Authors:** D. A. Skvortsov, I. V. Zhirkina, D. A. Ipatova, A. R. Pisarev, A. S. Malyshev, Y. A. Ivanenkov, V. G. Kartsev, O. A. Dontsova

**Affiliations:** 1 Chemistry Department and Belozersky Institute of Physico-Chemical Biology, Moscow State University, Moscow, Russia; 2 Faculty of Biology and Biotechnologies, Higher School of Economics, Moscow, Russia; 3 Hertsen Moscow Oncology Research Institute, Moscow, Russia; 4 Federal State Unitary Enterprise Dukhov Automatics Research Institute, Moscow, Russia; 5 InterBioScreen Ltd, Chernogolovka, Russia; 6 Skolkovo Institute of Science and Technology, Moscow, Russia; 7 Shemyakin–Ovchinnikov Institute of Bioorganic Chemistry, Russian Academy of Sciences, Moscow, Russia

**Keywords:** cytotoxicity, anticancer drugs, kinase inhibitors, FCCT test, selectivity

## Abstract

To search for substances selectively acting on tumor cells, phenotypic screening in a coculture of tumor cells with non-tumor cells was used in the work. The compound STOCK7S-36520, selectively cytotoxic in the coculture of breast tumor cells MCF7' and non-tumor MCF10A cells, contains structural elements characteristic of kinase inhibitors. Analysis of the compound STOCK7S-36520 and its derivative STOCK7S-47016 showed that they are new multikinase inhibitors. The highest inhibition of 84% was shown by compound STOCK7S-47016 against GCK kinase. Of interest is the significant selectivity of action against some of the cell lines studied: the selectivity index of STOCK7S-36520 against the prostate tumor cell line PC3 is 29 times compared to the model line of non-tumor fibroblasts VA13.

## INTRODUCTION

According to a review [1] by the Lancet Commission on Oncology, the search for low-molecular-weight drugs is one of the priority directions in the search for, development, and delivery of anticancer drugs. The two main experimental approaches to the initial selection of potential anticancer compounds are targeted and phenotypic searches. Targeted search is used when the biochemical pathway that is deregulated in pathology is known. In this case, screening is focused on one specific molecular mechanism (for a review, see [2]). Although the detected effect on a single molecular target may not reflect the overall biological activity of compounds, this approach is sufficiently effective [3]. The second approach is phenotypic—a search for compounds that affect the signs of pathology in a cellular model or in an organism. In this case, it is not necessary to know the molecular mechanism of the disease in detail. This approach has also  been successfully used to search for anticancer drugs [4].

The simplest desired phenotype for drug selection is the tumor cell death [5]. The key parameter in this case is the selectivity of the tested compounds, which is desirable to control from the early stages of drug development [6]. Co-cultivation of tumor and non-tumor cell lines allows immediately taking into account the specificity of action of the studied compound between the types of cocultured cells. In addition, it makes it possible to reveal compounds that do not show selectivity in monocultures [7, 8].

The compounds studied in this work contain structural elements characteristic of kinase inhibitors [9]. Many protein kinases regulate cell growth, proliferation, and differentiation, and mutations or changes in the expression of their genes can lead to uncontrolled growth and proliferation, becoming one of the drivers of tumorigenesis. Such protein kinases can be used as targets for the development of anticancer agents (for a review of existing drug molecules, see [10]).

The most common approach in the search for anticancer kinase inhibitors is to model kinase inhibitors and then test the most promising inhibitors in cellular and in vivo models. On the other hand, the majority of drugs were found using phenotype-based search [4]. With this in mind, based on preliminary data on the cytotoxicity of the compound STOCK7S-36520, we decided to investigate the selectivity of cytotoxicity in cell lines for it and its structurally related compounds, as well as to test the hypothesis that the most selective ones are kinase inhibitors.

## MATERIALS AND METHODS

The small molecule library was purchased from IBScreen. Molecule names are given according to vendor identifiers.

Human cell lines were grown at 37°C and 5% CO_2_. Lines A549, VA13, MCF7', HEK293T, HCT116, HepG2, PC3, SiHa, MDA-MB-231, and HT1080 were cultured in DMEM/F-12 medium (Paneko LLC, Russia) with 10% FBS (HiMedia, India). MCF7 line was cultured in DMEM/F-12 medium (Paneko LLC, Russia) with 10% FBS (INTL KANG, China). LNCaP line was cultured in RPMI medium (Paneko LLC, Russia) with 10% FBS (INTL KANG, China). U87 line was cultured in EMEM medium (Paneko LLC, Russia) with 10% FBS (INTL KANG, China). MCF10A line was cultured in 10A6+ medium (DMEM/F-12 supplemented with 5% horse serum, 20 ng/mL EGF, 0.5 mg/mL hydrocortisone (Thermo Fisher Scientific, United States), 100 nM (−)-isoproterenol hydrochloride (Sigma-Aldrich, United States), and 10 μg/mL insulin (Paneko LLC, Russia). All media were supplemented with 2 mM alanyl-glutamine (Paneko LLC, Russia), 50 U/mL penicillin, and 0.05 mg/mL streptomycin (Thermo Fisher Scientific, United States). HepaRG line was cultured in accordance with the recommendations of its developers.

To perform the FCCT test [8], cells of the lines expressing the EGFP or Katushka2S fluorescent proteins were co-seeded in 384-well plates (Greiner #781182). For the lung cancer model, A549_eGFP cells were seeded together with VA13_Kat cells at a rate of 400 and 800 cells, respectively, in F-12 medium with 10% FBS (HiMedia, India). For the breast cancer model, MCF7_eGFP and MCF10A_Kat cells were seeded in 384-well plates at a rate of 500 and 700 cells, respectively, in F-12 medium with 10% FBS (HiMedia, India) containing 20% 10A6+ medium. After culturing for 16–18 h, serial dilutions of the tested compounds were added to the cells: 50, 12.5, 2.5, 0.5, 0.125, and 0.025 mg/L. Then, the cells were incubated for 72 h at 37°C and 5% CO_2_. The plates were scanned with a TYPHOON FLA950 laser scanner (GE Healthcare, United States) at a a resolution of 10 μm. A 473-nm laser (600 V) with a 520–540 nm emission filter was used to detect eGFP, and a 635-nm laser (850 V) and a 665-nm filter were used to detect Katushka2S. The images of the plates were processed using the ImageJ editor according to [11], determining the number of survived cells by their fluorescence relative to control wells with cells without compounds [8].

MTT was carried out as described in [12]. VA13 or MCF7 cells (4000 cells per well), or HEK293T cells (2500 cells per well), or a precultured monolayer of differentiated HepaRG cells, or 3000 cells per well for the remaining cell lines were seeded in 96-well plates with culture medium. After incubation for 18 h, the compounds diluted in the medium were added (eight concentrations with a 3x step, the highest concentration in the cells was 0.05 mg/mL). Incubation was performed for 72 h at 37°C and 5% CO_2_. Then, MTT was added to a concentration of 0.5 mg/mL, and incubation was carried out for 2–4 h at 37°C and 5% CO2. Thereafter, MTT solution was removed, and 140 μL of DMSO per well was added. Optical density was measured using a plate photometer at a wavelength of 565 nm. The results were processed using GraphPad software (San Diego, California).

The mechanism of cell death was analyzed by flow cytometry with Annexin-Alexafluor488 and propidium iodide (Thermo Fisher) according to the manufacturer’s recommendations.

Prediction of kinases as possible targets of the compounds was carried out using SEA (sea.bkslab.org), PassTargets (www.way2drug.com/passtargets/), Swisstargetprediction (swisstargetprediction.ch/), and Golden Cubes (pharma.ai/chemistry42#rec821652551) services.

Kinase inhibition assays were performed according to the KinaseProfiler protocol.

## RESULTS AND DISCUSSION

**Fig. 1.**
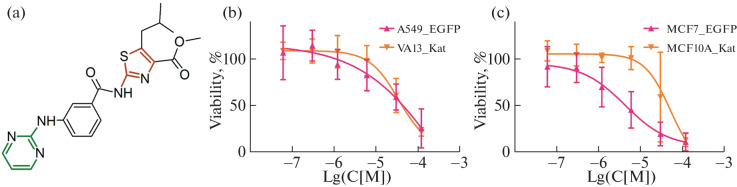
(a) The structure of compound STOCK7S-36520, the pyrimidine-2-amine fragment is shown in green, the amino-2-thiazole fragment is shown in beige. (b, c) Cell survival rates as a function of the drug concentration in the test in cocultures of A549'_EGFP with VA13_Kat and MCF7'_EGFP with MCF10A_Kat, respectively. The dependences for tumor and etiologically non-tumor cells are shown in red and yellow, respectively.

The FCCT test [8] was used to screen the compounds in cocultures of tumor and non-tumor cells. To assess the selectivity of action, the number of successive dilutions of the compounds was determined, during incubation with which the survival of the conditionally normal cell line was twice or more higher than the survival of tumor cells. During a single-repeat screening, the STOCK7S-36520 compound at one of the dilutions showed selective cytotoxicity against the lung tumor cells A549'_EGFP cocultured with the etiologically non-tumor fibroblasts VA13_Kat. This compound also selectively suppressed the growth of the breast tumor cells MCF7'_EGFP cocultured with the non-tumor cells MCF10A_Kat in two successive dilutions. A more detailed analysis (five replicates) of this compound showed that selectivity in the coculture of A549'_EGFP with VA13_Kat was manifested only in some of the replicates, whereas the effect on MCF7'_EGFP in the coculture with MCF10A_Kat was reproducible. The structure of the STOCK7S-36520 compound and cell survival data are shown in Fig. 1.

**Fig. 2.**
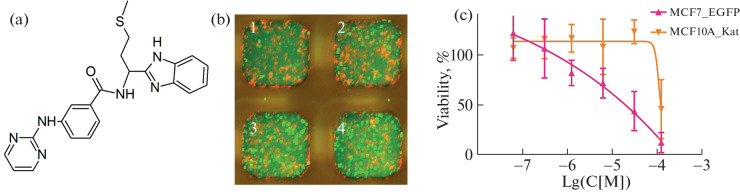
(a) The structure of STOCK7S-47016. (b) An example of an image of cells with tumor cells MCF7'_EGFP (green) in coculture with non-malignant MCF10A_Kat (red) during high-resolution scanning in the FCCT test. 1—50 μg/mL STOCK7S-47016; 2—12.5 μg/mL STOCK7S-47016; 3—2.5 μg/mL STOCK7S-47016; 4—without the drug. (c) FCCT test results for the drug STOCK7S-47016 in the FCCT test in the coculture of breast cells, the data for tumor and non-tumor cells are shown in red and yellow, respectively.

No data on the cytotoxicity of STOCK7S-36520 or its effect on a specific molecular target were found in the Pubchem and SciFinder databases, although the group of its homologues includes compounds mentioned in patents devoted to tyrosine kinase inhibitors [13]. Analysis of the structure of its molecule revealed two regions that may be responsible for kinase inhibition: the amino-2-thiazole fragment [14] and the pyrimidine-2-amine fragment [9] (Fig. 1a). In addition, the cytotoxic effect on cells may be due to other substituents and mechanisms. For this reason, a mini-library of 95 compounds including one or both putative key fragments of STOCK7S-36520 was assembled and studied in both cocultures. The FCCT screening data for the most selective compounds are presented in the first two columns of Table 1. The highest selectivity of cytotoxicity in the screening was found for the compound STOCK7S-47016 (Fig. 2).

**Table 1.** Evaluation of the effect of stock7S-36520 and a series of compounds with similar substructural fragments in cocultures of cell lines and individual cell lines. N (A549/VA13) and N (MCF7'/MCF10A) are the numbers of serial dilutions in cocultures of A549_EGFP with VA13_Kat and MCF7'_EGFP with MCF10A_Kat, respectively, at which the difference in survival was 2 or more times; medians of 2–5 replicates are given. IC_50abs_ are the concentrations that cause half-survival of cells in the corresponding monocultures in the MTT test. SIs are the selectivity indices calculated based on the MTT test data.

ID	N(A549/VA13)	N(MCF7'/MCF10A)	IC50abs, mg/ L (HEK293T)	IC_50abs_, mg/ L (MCF7)	IC_50abs_, mg/ L (VA13)	IC_50abs_, mg/ L (A549)	SI (VA13/MCF7')	SI (VA13/ A549)
STOCK7S-47016	0.5	2.5	0.17 ± 0.01	2.28 ± 0.55	2.55 ± 0.44	1.16 ± 0.17	1.1	2.2
STOCK7S-28375	0	2.5	0.21 ± 0.02	2.54 ± 0.45	0.89 ± 0.17	0.67 ± 0.11	0.4	1.3
STOCK7S-36520	0.5	2	0.40 ± 0.04	9.7 ± 4.25	25.44 ± 2.22	5.97 ± 1.99	2.6	4.3
STOCK2S-60514	0	2	0.78 ± 0.06	1.16 ± 0.46	0.72 ± 0.18	2.48 ± 1.09	0.6	0.3
STOCK6S-48386	1.5	0	3.64 ± 0.13	4.63 ± 0.55	4.08 ± 0.31	4.76 ± 0.34	0.9	0.9
STOCK2S-29680	0	1.5	4.15 ± 0.52	26.37 ± 5.62	36.97 ± 7.92	49.64 ± 6.87	1.4	0.7
STOCK7S-27720	0	1.5	5.32 ± 0.31	6.55 ± 0.62	14.09 ± 1.68	54.68 ± 5.89	2.2	0.3
STOCK7S-40584	0.5	1.5	7.46 ± 0.58	8.47 ± 1.26	8.47 ± 0.98	39.77 ± 2.48	1.0	0.2

**Fig. 3.**
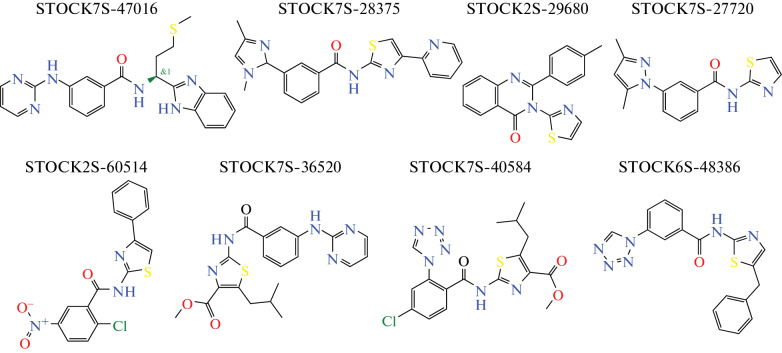
Structures of the compounds considered in Table 1.

For the eight compounds with the highest selectivity of action shown in the screening in cocultures of A549'_EGFP with VA13_Kat and MCF7'_EGFP with MCF10A_Kat (structures are shown in Fig. 3), cytotoxicity assays in monocultures were performed (Table 1, IC_50abs_). Cytotoxicity was determined by the classical method according to Mossman (MTT) using the human breast cancer cell line MCF7, the human epithelial lung carcinoma cell line A549, and the non-cancer lung fibroblast cell line VA13. Cytotoxicity was also assessed for the immortalized human embryonic kidney cell line HEK293T as the fastest growing cell line in our cell line panel.

**Fig. 4.**
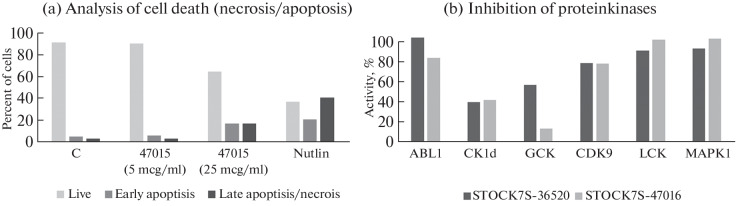
Analysis of possible mechanisms of drug action on cells. (a) Induction of cell death by the compound STOCK7S-47016 (necrosis/apoptosis in A549). (b) Inhibition of protein kinases by the compounds STOCK7S-36520 and STOCK7S-47016 at 10 μM of the compound and ATP, the activity of kinases normalized to the sample without the inhibitor is shown.

The selectivity of both the original molecule STOCK7S-36520 and the compound STOCK7S-47016, when comparing their action on tumor cell lines with the non-tumor fibroblast model, was low and was observed predominantly against A549 lung cancer cells. Therefore, the cell death (necrosis/apoptosis) assay for the compound STOCK7S-47016 was performed on A549 cells. It was found that this compound can induce apoptosis (Fig. 4a).

In addition, all three most selective compounds (Table 1) were more potent in rapidly dividing HEK293T cells. Therefore, the selectivity of the STOCK7S-36520 and STOCK7S-47016 molecules was analyzed in an expanded set of cell lines. Treatment with these compounds resulted in the death of PC3 cells at much lower concentrations than VA13 cells (Table 2).

**Table 2.** Cytotoxicity (IC_50abs_) and selectivity indices towards VA13 line (SI) of compounds STOCK7S-36520 and STOCK7S-47016 in MTT analysis in ten cell lines

Cell line	IC50abs (STOCK7S-36520), mg/L	SI (STOCK7S-36520)	IC50abs (STOCK7S-47016), mg/L	SI (STOCK7S-47016)
VA13	2.93 ± 0.33	1	13.88 ± 4.64	1
HEPG2	0.51 ± 0.03	5.7	17.38 ± 5.09	0.8
LNCaP	0.74 ± 0.14	4	6.25 ± 1.95	2.2
U87	1.59 ± 0.18	1.8	6.64 ± 0.83	2.1
HT1080	2.03 ± 0.17	1.4	19.89 ± 5.18	0.7
PC3	0.1 ± 0.02	29.3	1.74 ± 0.31	8
MDA-MB-231	3.12 ± 0.46	0.9	1.7 ± 0.26	8.2
SiHa	1.44 ± 0.11	2	15.67 ± 2.85	0.9
HCT116	0.39 ± 0.05	7.5	5.51 ± 0.92	2.5
HepaRG	6.8 ± 3.26	0.4	7.54 ± 3.65	1.8

To identify the possible mechanism of action of compounds STOCK7S-36520 and STOCK7S-47016, their effect on the activity of six kinases, putative molecular targets, was studied. It turned out that both compounds inhibited the activity of casein kinase CK1d by more than 50%, and STOCK7S-47016 inhibited the activity of GCK kinase by 86% (Fig. 4b).

The majority of chemotherapeutic drugs have significant side effects, including due to their toxicity to dividing normal cells. The most important requirement for such new small molecules is to reduce their side effects [6]. In addition, analysis in cell models is almost always one of the important steps in the development of compounds against tumor cells. For this reason, in this work we used the recently developed FCCT test. It allowed us to detect selective cytotoxicity of the STOCK7S-36520 molecule and several structurally related compounds in the coculture of fluorescently labeled MCF7 and MCF10A cells.

The compounds identified using the FCCT may exhibit greater selectivity in cell lines other than those used in the initial screening. For example, compound 65D08 [8], which had an effect in a pair of serial dilution in coculture in the expanded set of cell lines, had an SI of approximately 100 against the specific prostate cancer cell line LNCAP in the expanded cytotoxicity assay compared to VA13 [15]. In the case of compounds STOCK7S-36520 and STOCK7S-47016, the highest selectivity (SI = 29) was exhibited against the PC3 cell line compared to VA13, rather than against the lines from the initial cocultures. The spectrum of activity of this pair of compounds on cell lines somewhat differed. The HCT116 line was the second most sensitive line to the STOCK7S-36520 compound, similarly to PC3 growing in our conditions significantly faster than the reference line of etiologically non-tumor fibroblasts VA13. At the same time, compound STOCK7S-36520 showed the lowest cytotoxicity against the model of normal hepatocytes HepaRG. In the case of STOCK7S-47016, the line that ranked second in sensitivity to it was MDA-MB-231, which, similarly to MCF7 line in the screening, had a breast pathology origin.

Further developments using these molecules require an analysis of their mechanisms of action on the cell. The biological activity of the compounds containing substructural elements known in the databases can be predicted. When analyzing the possible molecular targets for the compounds in the PASStargets, SwissTargetPrediction, and SEA (Similarity Ensemble Approach) services, the main pool of target proteins belonged to kinases. The STOCK7S-36520 molecule contains aminothiazole and pyrimidine-2-amine fragments, which may be responsible for kinase inhibition [9, 14] (Fig. 1a). Structural elements similar to STOCK7S-36520 and STOCK7S-47016 are found in the well-known kinase inhibitors nilotinib and imatinib. Based on the data for the studied molecules from the bioactivity prediction services and the published data on their known homologues [16, 17], we selected several kinases for the initial assessment of their activity. A significant decrease in activity as a result of the action of the compounds was observed for the casein kinase CK1d (CSNK1d) and GCK kinase (MAP4K2). It should be noted that GCK kinase individually is not essential for the survival of cell lines according to the Depmap project [18], although it significantly reduces the viability of some lines. According to our data, the STOCK7S-47016 compound can induce apoptosis (Fig. 4a). Therefore, it can be assumed that the effect of these compounds on cells is multitarget.

## CONCLUSIONS

Compounds STOCK7S-36520 and STOCK7S-47016, which exhibit selective cytotoxicity in the coculture of breast tumor cells MCF7 and non-tumor cells MCF10A, are multikinase inhibitors containing known substructural elements characteristic of kinases, but not previously described as kinase inhibitors. The greatest inhibition of activity (84%) was found for compound STOCK7S-47016 against GCK kinase. Of interest is the significant selectivity of the studied compounds against the fast-growing cell lines, in particular, STOCK7S-36520 against the prostate tumor cell line PC3, for which the selectivity index compared to the model line of non-tumor fibroblasts VA13 is 29 times. Study of the mechanism of interaction of these compounds with kinases will be the subject of further studies.
